# Nomogram for predicting pregnancy-related relapse of myasthenia gravis

**DOI:** 10.1186/s13023-024-03466-6

**Published:** 2024-12-01

**Authors:** Manqiqige Su, Xiaoqing Liu, Zongtai Wu, Jie Song, Xiao Huan, Huahua Zhong, Rui Zhao, Chongbo Zhao, Yali Zhang, Sushan Luo

**Affiliations:** 1 Huashan Rare Disease Centre and Department of Neurology, Huashan Hospital, Shanghai Medical College, National Centre for Neurological Disorders, Fudan University, Shanghai, 200040 China; 2 Department of Neurology, Chifeng Municipal Hospital, Chifeng, China; 3 Department of Public Health and Primary Care, University of Cambridge, Cambridge, CB2 0BB UK

**Keywords:** Myasthenia gravis, Pregnancy, Thymectomy, Risk factors, Nomogram

## Abstract

**Background:**

Myasthenia gravis (MG) is an autoimmune disease mediated by autoantibodies primarily affecting the neuromuscular junction. This study aims to identify risk factors for pregnancy-related MG relapse and develop a predictive model to improve clinical outcomes.

**Methods:**

We enrolled 113 MG female patients with a pregnancy history during follow-up at Huashan Hospital affiliated with Fudan University, between January 2015 and October 2021. The study analyzed relapse rates and risk factors during pregnancy and postpartum using multivariate logistic regression. A nomogram was constructed to predict relapse probability, with model performance evaluated by discrimination and calibration metrics.

**Results:**

Of the 113 patients, 52 (46.02%) experienced 115 relapses, including 52 (45.22%) occurring during the first trimester of pregnancy, 11 (9.56%) during the second trimester of pregnancy, and 52 relapses (45.22%) during the three months after delivery/abortion. Significant factors associated with pregnancy-relate relapse, included age at delivery/abortion (OR 0.21, 95% CI 0.06–0.65), MG stable duration (OR 0.24, 95% CI 0.09–0.63), thymic hyperplasia (OR 3.45, 95% CI 1.35–9.3), pre-pregnancy thymectomy (OR 0.08, 95% CI 0.01–0.36), and inadequate treatment during pregnancy (OR 4.44, 95% CI 1.35–17.76). The Nomogram model demonstrated robust predictive performance.

**Conclusion:**

The first trimester of pregnancy and three months following delivery or abortion are high-risk periods for MG relapse. Younger ages, shorter MG stable duration before pregnancy, thymic hyperplasia, and inadequate treatments during pregnancy increase relapse risk.

## Introduction

Myasthenia gravis (MG) is a chronic autoimmune disorder that impacts the neuromuscular junction (NMJ), resulting in skeletal muscle weakness. It is primarily mediated by autoantibodies, including the ones targeting the acetylcholine receptor (AChR), muscle-specific tyrosine kinase (MuSK), and low-density lipoprotein receptor-related protein 4 (LRP4). Incidence and prevalence of MG vary across groups of different ages, genders, and ethnicities [1]. On a global scale, the incidence of MG is estimated to range from 3 to 30 cases per million person-years, while its prevalence ranges from 150 to 200 cases per million [2, 3]. In China, the incidence of MG is reported at 6.8 cases per million person-years, with an admission mortality rate of 14.69 ‰. Respiratory failure and lung infection are the leading causes of mortality in these patients [4].

Gender disparities in MG are pronounced, with women exhibiting higher prevalence and incidence rates compared to men. The female-to-male ratio of MG patients is generally around 2:1, though this ratio can vary depending on age and disease subtypes [5, 6]. The onset of MG demonstrates a bimodal age distribution, with the first peak occurring between 20 and 40 years old [7, 8]. This period coincides with the prime childbearing years, yet the effect of pregnancy on MG symptom management is highly variable and remains challenging to predict.

Pregnancy-related relapse rates of MG have been reported to range from 19 to 50%, with symptom exacerbation often occurring during the early stages of pregnancy or in the postpartum period following delivery or abortion [9–11]. Previous studies have identified potential factors contributing to these relapses, including increased gestation BMI gain [12], shorter disease duration, and discontinued immunosuppressive treatment [13]. Despite these findings, the majority of existing research is descriptive, and the precise risk factors for pregnancy-related MG relapses of MG remain inadequately understood. Furthermore, there is a lack of sufficient cohort studies on MG patients during pregnancy in China.

Therefore, we conducted a retrospective study to gain a deeper understanding of the impact of pregnancy on MG outcomes. Our study aimed to identify the risk factors associated with pregnancy-related MG relapses and to develop a nomogram model to predict relapse probability. The findings of this study are expected to inform more effective management strategies for pregnant women with MG and improve clinical outcomes for this patient population.

## Materials and methods

### Study population and sample collection

We retrospectively collected data from 513 female MG patients who have been registered at Huashan Hospital affiliated with Fudan University between January 2015 and October 2021. The inclusion criteria of our study were: (a) female patients; (b) aged between 18 and 45 at the time of registration; and (c) diagnosed with MG based on the Myasthenia Gravis Foundation of America (MGFA) criteria before pregnancy [14]. The exclusion criteria included: (a) the presence of systemic diseases or conditions such as congenital myasthenic syndrome, Lambert-Eaton syndrome and botulinum toxin poisoning; (b) severe immunodeficiency; (c) history of mental illness; and (d) lack of regular follow-up or incomplete baseline information.

**Fig. 1:**
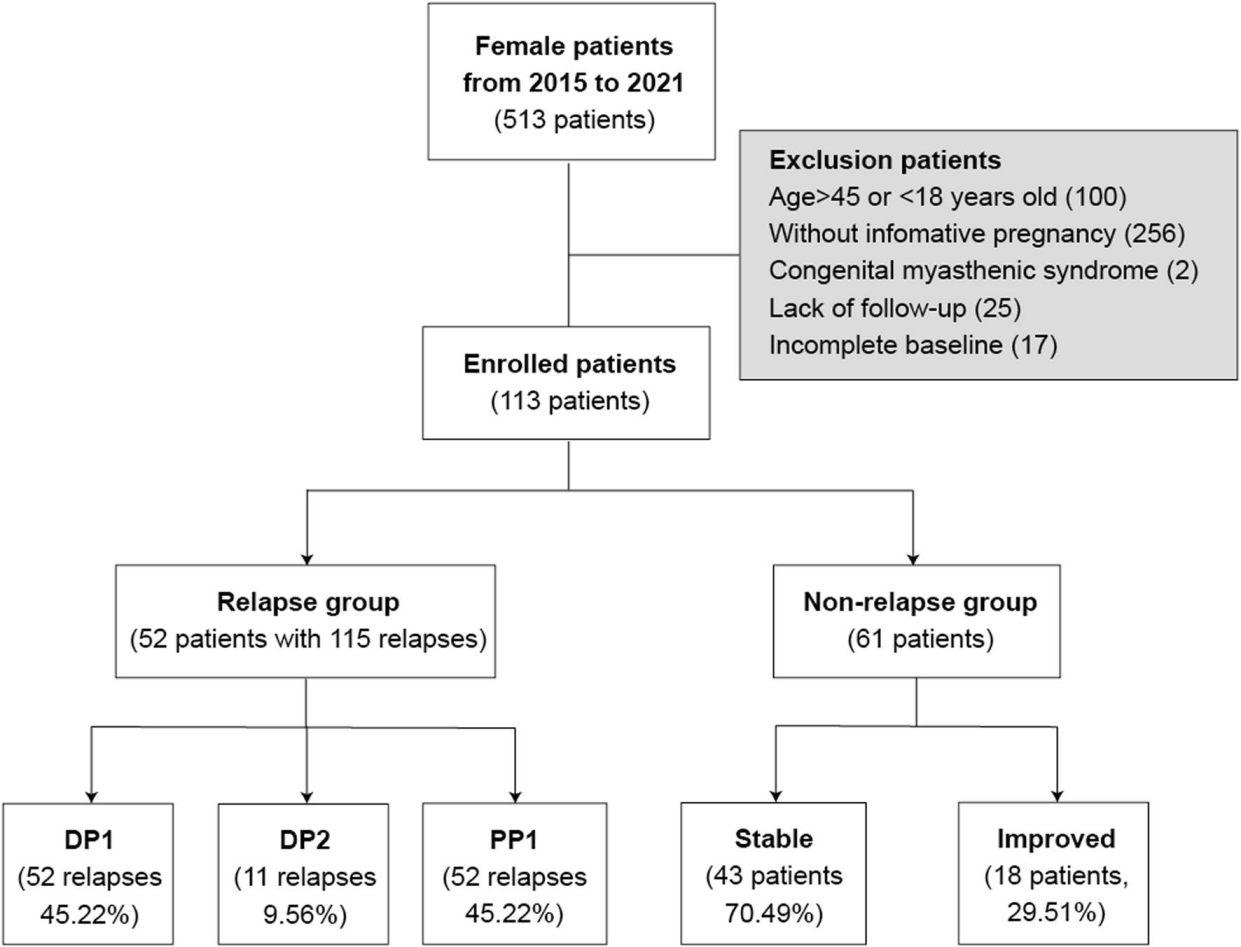
Study flowchart of the enrolled patients. DP1, 0–3 months during pregnancy; DP2, 4–6 months during pregnancy; PP1, 0–3 months of the postpartum period

Ultimately, we enrolled 113 MG female patients with a pregnancy history during the follow-up period (Fig. 1). Based on changes in the MG Activities of Daily Living (ADL) scores (increased MG-ADL score ≥ 2 compared to the previous stage defined as the MG relapse), patients were categorized into the relapse group (*n* = 52) and the non-relapse group (*n* = 61). The patient’s MG Quality of Life (QOL15) scores were also retrospectively analyzed [15, 16].

To assess the MG outcome during the pregnancy, we subsequently divided the pregnancy period into the following stages: before pregnancy (BP), 0–3 months during pregnancy (DP1), 4–6 months during pregnancy (DP2), 7–9 months during pregnancy (DP3), 0–3 months of the postpartum period (PP 1), 4–6 months of the postpartum period (PP 2), and 7–12 months of the postpartum period (PP 3 + 4).

### Definitions and diagnosis standards

Treatment in 6 months before pregnancy, during pregnancy and after delivery/abortion were classified into inadequate and adequate treatments. Inadequate treatment was defined as meeting at least one of the following conditions: (a) no treatment at all; (b) low-dose oral prednisone (≤ 10 mg/day) or cholinesterase inhibitors used as monotherapy. The adequate treatment referred to: (a) usage of relatively high doses of oral prednisone (> 10 mg/day) in combination with cholinesterase inhibitors; (b) use of immunosuppressants with or without oral prednisone; (c) usage of rituximab 6 months before pregnancy or shortly after delivery/abortion, at the recommended dose for monotherapy (375 mg/m^2^); (d) undergoing plasmapheresis (PE) and intravenous immunoglobulin (IVIG) treatment before and during pregnancy [17]. A best practice guideline from a U.K. multispecialty working group recommended the use of prednisone as normal but “lowest effective dose” [17]. We usually suggest keeping the corticosteroids after pregnancy at a minimum dose to maintain an MSE status. The oral corticosteroid was meant to be maintained till the three months postpartum. In our cohort, most participants were on prednisone of 10 mg/day as maintenance therapy, thus we use this dose as the cut-off value. Diagnostic criteria for abnormal blood glucose and blood pressure followed the guidelines for managing pregnancy complicated by diabetes and hypertension [18–20].

### Statistical analysis

Categorical variables were summarized as proportions and analyzed using the chi-square or Fisher’s exact test, as appropriate. The Shapiro–Wilk normality test was applied to all continuous parameters to test for normality. If the test values were below the significance threshold (*P* = 0.05), the median and interquartile (IQR) range were used for the descriptive characteristics; otherwise, the mean and standard deviation (SD) were used to describe the data. Normally distributed continuous variables were analyzed using the t-test, while the non-normally distributed continuous variables were analyzed using the Mann–Whitney U test. A *P* value < 0.05 is considered of significance. Statistical analysis was performed using IBM SPSS Statistics software (v 27.0) (IBM, Armonk, NY, USA). Chart making was performed using R (v 4.1.1) or GraphPad Prism (v 8.0).

The association between maternal variables and pregnancy-related relapses was determined using univariate and multivariate logistic regression. To identify the risk factors for an unfavorable outcome, univariate (statistical significance, α = 0.2) and multivariate (statistical significance, α = 0.05) logistic regression analysis with an odds ratio (OR) was performed. Variables that did not reach the levels of statistical significance (*P* = 0.05) were eliminated and a new multivariate model was set. The predictive model of pregnancy-related relapse with risk factors was depicted in a nomogram using R (v 4.1.1).

The discrimination performance of the nomogram was measured using the area under the receiver operating characteristic curve (AUC‐ROC) in the derivation cohort, with 95% confidence intervals (95% CI) reported. Calibration was evaluated using the Hosmer–Lemeshow goodness‐of‐fit test and calibration plots. A two‐tailed *P* < 0.05 was considered significant.

## Results

### Pregnancy outcomes and pregnancy-related relapses

Among the 513 female MG patients being screened, a total of 113 pregnancies were collected from 113 patients with the following outcomes: 29 (25.66%) full-term vaginal deliveries, 50 (44.25%) full-term cesarean sections, 3 (2.65%) preterm deliveries, 27 (23.89%) elective terminations, and 4 (3.54%) spontaneous abortions. The distribution of these obstetric outcomes did not demonstrate a statistically significant difference between the relapse and non-relapse groups (*P* = 0.062).

Before pregnancy, all MG patients had achieved a stable state with an ADL score of less than 2 points (*n* = 113). During pregnancy or within one year postpartum, 52 patients (46.02%) experienced an MG relapse with an ADL score increased more than 2 points, whereas 61 patients (54.98%) did not. Within the non-relapse group, 43 patients maintained a stable state, and 18 patients exhibited improvement compared to their pre-pregnancy condition. In the relapse group, 52 patients experienced a total of 115 relapses: 52 (45.22%) during DP1,11 (9.56%) during DP2, and 52 (45.22%) during PP1.

### MG-ADL and MG-QOL15 score in each phase of pregnancies

**Fig. 2:**
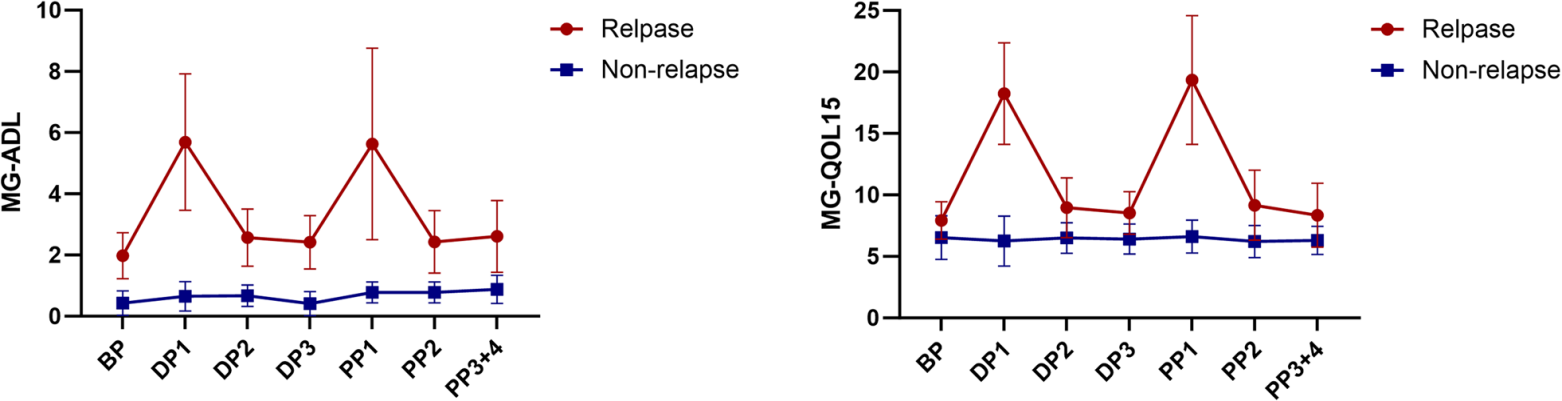
Changes in MG-ADL and MG-QOL15 scores of relapse group and non-relapse group at different stages of the pregnancy. BP, before pregnancy; DP1, 0–3 months during pregnancy; DP2, 4–6 months during pregnancy; DP3, 7–9 months during pregnancy; PP1, 0–3 months of the postpartum period; PP2, 4–6 months of the postpartum period; PP3 + 4, 7–12 months of the postpartum period

Changes in MG-ADL and MG-QOL15 scores in patients at different stages are shown in Fig. 2. In the relapse group, the average scores of MG-ADL and MG-QOL15 in the DP1 and PP1 stages were significantly higher compared to those in the BP (*P* < 0.05). However, in the non-relapse group, there were no significant differences in MG-ADL and MG-QOL15 scores at any stage compared to BP (*P* > 0.05). At the same gestational stages, the MG-ADL and MG-QOL15 scores in the relapse group were significantly higher than those in the non-relapse group (*P* < 0.05).

### Clinical features of relapse and non-relapse groups

The clinical characteristics of patients in the relapse and non-relapse group were analyzed (Table 1). In this cohort, we included patients with a mean onset age of 26.08 ± 2.39 years including 36 ocular MG (OMG) cases (31.86%) and 77 GMG cases (68.14%). The participants enrolled in this cohort have concurrent rheumatoid diseases and other autoimmune diseases, such as rheumatoid arthritis (*n* = 11), systemic lupus erythematosus (SLE) (*n* = 1), and Hashimoto’s thyroiditis (*n* = 7). Among the 21 patients who had adequate treatment before pregnancy, the use of IS was reported in 10 patients, rituximab was applied in 4 patients and PE/IVIG treatments in 5 patients. In contrast, among the 30 patients who had adequate treatment during pregnancy, 1 patient used IS and 6 patients had PE/IVIG for controlling symptom fluctuations.

**Table 1:** Clinical characteristic between relapse and non-relapse groups

Features	Total (*n* = 113)	Relapse group (*n* = 52)	Non-relapse group (*n* = 61)	P value
Onset age (years old), mean ± SD	26.08 ± 2.39	25.9 ± 2.45	26.23 ± 2.34	0.473
Delivery/Abortion age, n (%)				**0.021**
20 ~ 35	84 (74.34)	44 (84.62)	40 (65.57)	
36 ~ 45	39 (34.51)	8 (15.38)	21 (34.42)	
MG stable duration, n (%)				**0.001**
< 2 years	61 (53.98)	37 (71.15)	24 (39.34)	
≥ 2 years	52 (46.02)	15 (28.85)	37 (60.66)	
MGFA classification, n (%)				0.214
I	54 (47.79)	24 (46.15)	30 (49.18)	
IIa	46 (40.71)	19 (36.54)	27 (44.26)	
IIb	10 (8.85)	7 (13.46)	3 (4.92)	
IIIa	2 (1.77)	1 (1.92)	1 (1.64)	
IIIb	1 (0.88)	1 (1.92)	0	
MG subtype, n (%)				0.861
OMG	36 (31.86)	17 (32.69)	19 (31.15)	
GMG	77 (68.14)	35 (67.31)	42 (68.85)	
Serum subtype, n (%)				0.075
AChR-Ab	73 (64.4)	33 (63.46)	40 (65.57)	
MuSK-Ab	22 (19.47)	14 (26.92)	8 (13.11)	
Negative	18 (15.93)	5 (9.62)	13 (21.31)	
Thymic hyperplasia, n (%)	42 (37.17)	28 (53.85)	14 (22.95)	**0.001**
Thymoma, n (%)	9 (7.96)	7 (13.46)	2 (3.28)	**0.046**
Thymectomy, n (%)	21 (18.58)	5 (9.62)	16 (26.23)	**0.024**
Thyroid dysfunction, n (%)	21 (18.58)	7 (13.46)	14 (22.95)	0.196
MC history before pregnancy, n (%)	8 (7.08)	5 (9.62)	3 (4.92)	0.547
Hyperglycemia, n (%)	20 (17.7)	11 (21.15)	9 (14.75)	0.374
Hypertension, n (%)	29 (25.66)	10 (19.23)	19 (31.15)	0.148
Treatment before pregnancy, n (%)				0.747
Inadequate	92 (81.42)	43 (82.69)	49 (80.32)	
Adequate	21 (18.58)	9 (17.31)	12 (19.67)	
Treatment during pregnancy, n (%)				**0.001**
Inadequate	83 (73.45)	33 (63.46)	50 (81.97)	
Adequate	30 (26.55)	19 (36.54)	11 (18.03)	

MG, myasthenia gravis; OMG, ocular myasthenia gravis; GMG, general myasthenia gravis; MC, myasthenia crisis; AChR, acetylcholine receptor; MuSK, muscle-specific tyrosine kinase

P-values less than 0.05 are highlighted in bold

Compared to the non-relapse group, the relapse group had a significantly higher proportion of patients who received adequate treatment during pregnancy and perinatal period (81.97% vs 63.46%, *P* = 0.001), as well as a higher proportion of patients with a stable disease duration of fewer than two years (71.15% vs. 39.34%, *P* = 0.001). Additionally, the incidence of thymic hyperplasia (53.85% vs. 22.95%, *P* = 0.001) and thymoma (13.46% vs. 3.28%, *P* = 0.046) was significantly higher in the relapse group. Conversely, the proportion of patients who had undergone thymectomy prior to pregnancy was significantly lower in the relapse group (9.62% vs. 26.23%, *P* = 0.024), and the age at pregnancy was significantly lower (*P* = 0.021).

### Risk factors and the prediction model of pregnancy-related relapse

To investigate the risk factors of pregnancy-related relapse, we conducted univariate and multivariate logistic regression calculations on clinical factors with significant differences between the two groups in the previous analyses (Table 2). The results ultimately identified the following as significant factors associated with pregnancy-related MG relapse: age at delivery/abortion (OR 0.21, 95% CI 0.06 – 0.65), duration of disease before pregnancy (OR 0.24, 95% CI 0.09 – 0.63), thymic hyperplasia (OR 3.45, 95% CI 1.35 – 9.3), pre-pregnancy thymoma surgery (OR 0.08, 95% CI 0.01 – 0.36), and inadequate treatment during pregnancy (OR 4.44, 95% CI 1.35 – 17.76). In summary, younger age at pregnancy, shorter disease duration, and the presence of thymic hyperplasia are risk factors for pregnancy-related MG relapse, whereas adequate treatment during pregnancy and pre-pregnancy thymectomy are protective factors, contributing to improved disease control.

**Table 2:** Risk factors of pregnancy-related relapse using univariate and multivariate logistic regression

Variable	Univariate analysis		Multivariate analysis	
	OR (95% CI)	*P* value	OR (95% CI)	*P* value
Delivery/Abortion age				
20 ~ 35	Ref.		Ref.	
36 ~ 45	0.35 (0.13 ~ 0.86)	**0.027**	0.21 (0.06 ~ 0.67)	**0.012**
Thyroid dysfunction	0.52 (0.18 ~ 1.38)	0.201		
Serum subtype, n (%)				
AChR-Ab	Ref.		Ref.	
MuSK-Ab	2.12 (0.81 ~ 5.89)	**0.134**	3.2 (0.91 ~ 12.88)	0.082
Negative	0.47 (0.14 ~ 1.38)	**0.186**	0.46 (0.1 ~ 1.9)	0.298
Hypertension	0.8 (0.31 ~ 1.98)	0.63		
MG stable duration				
< 2 years	Ref.		Ref.	
≥ 2 years	0.26 (0.17 ~ 0.57)	**0.0009**	0.24 (0.85 ~ 0.63)	**0.005**
Thymic hyperplasia	3.92 (1.77 ~ 8.99)	**0.0009**	2.91 (1.12 ~ 7.94)	**0.031**
Thymoma	4.589 (1.05 ~ 31.79)	**0.065**	4.29 (0.77 ~ 34.66)	0.166
Thymectomy	0.28 (0.09 ~ 0.76)	**0.019**	0.08 (0.01 ~ 0.36)	**0.002**
Treatment during pregnancy				
Adequate	Ref		Ref	
Inadequate	2.62 (1.12 ~ 6.37)	**0.029**	3.94 (1.25 ~ 14.15)	**0.024**

P-values less than 0.2 in the univariate analysis and less than 0.05 in the multivariate analysis are highlighted in bold

**Fig. 3:**
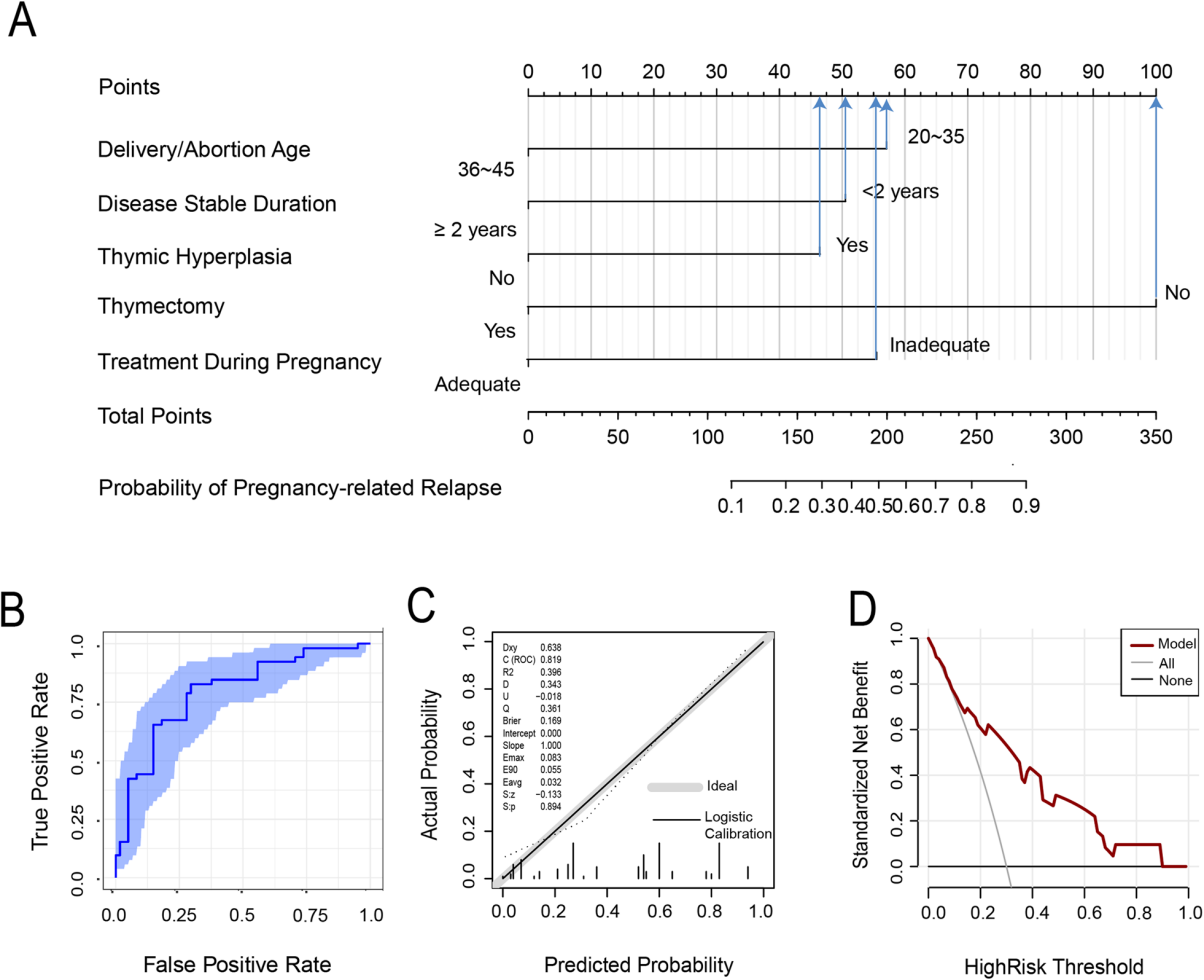
Nomogram to predict the risk of pregnancy-related relapse. AUC, area under curve. **A** The nomogram model to predict the risk of pregnancy-related relapses in MG patients; **B** ROC curve of bootstrap: the area under the curve (AUC) index was 0.82, indicating good prediction ability of the model. **C** Calibration curve: The *P*-value was 0.894, indicating good calibration of the nomogram model. **D** Decision curve analysis: Grey and black line represented results for “intervention for all” and “intervention for none”. Red line indicated the prediction model. Within the risk threshold of 10 – 90%, the prediction curve was above the “none” line and the “all” line, indicating the clinical practicability of the models

To illustrate the predictive model of pregnancy-related MG relapse, we developed a nomogram model (Fig. 3A), which exhibited strong discrimination and calibration. The nomogram demonstrated a C-index of 0.819, with a Hosmer–Lemeshow good of fit test chi-square value of 5.42 and a *P* value of 0.6084. The ROC curve of the nomogram is shown in Fig. 3B, indicating an area under the curve (AUC) index of 0.819 (95% CI 0.74 ~ 0.89). The calibration curve and decision curve analysis are presented in Fig. 3C and D respectively, with the calibration curve’s *P* value of 0.894, indicating good calibration of the nomogram model. Within the risk threshold of 10% to 90%, the prediction curve in the decision curve analysis was above the “none” line and the “all” line, demonstrating the clinical practicability of the model. The online calculator based on this nomogram model is available at https://mgresearch.shinyapps.io/MGpregnancy/, allowing prediction of pregnancy-related MG relapses by inputting the identified parameters.

## Discussion

In this study, the majority of pregnant patients with MG experienced relapses during the early stage of pregnancy (DP1) and within the first 0–3 months after delivery or abortion (PP1), which is consistent with previous research results [21]. Several factors such as hormone fluctuation, may contribute to this increased risk. During the early stage of pregnancy, the body undergoes an inflammatory phase caused by embryo implantation and placenta formation, which predisposes MG patients to relapse. After delivery or abortion, the concentrations of estrogen, progesterone and glucocorticoids drop sharply, while prolactin levels continue to rise. This hormonal shift leads to a pro-inflammatory environment, with increased pro-inflammatory response of T helper cell type 1 (Th1) and decreased anti-inflammatory response of regulatory T cells (Treg) [22–24]. Additionally, the physical exertion of childbirth or abortion, along with emotional stress and the demands of breastfeeding, further exacerbate the risk of MG relapses. In contrast, patients tend to be more stable in the second and third trimesters of pregnancy. Some studies suggest this may be associated with decreased leukocyte chemotaxis and adhesion during these periods, which may attenuate autoimmune activity [25]. In addition, other studies proposed that elevated alpha-fetoprotein levels during pregnancy act as inhibitors of AChR-Ab, thereby inducing an immunosuppressive anti-inflammatory response [26].

Noticeably, our study’s results are not concordant with the findings by Zhou Q, et al. [12]. The sample size difference in the two independent cohorts was part of the reason. In addition, the patients in our cohort have a younger onset age of 26.08 years and a relatively low thymectomy rate (18.6% vs. 21.6%). The duration from onset to conception may be longer in the previous cohort, which is 8 years. This explains the high proportion in our cohort of MG relapse associated with pregnancy (46.02%).

The regression analysis indicated that the younger age (ages 20 ~ 35) at delivery/abortion and a shorter period of disease stability before pregnancy are associated with an increased risk of MG relapse related to pregnancy. According to previous studies, this age group of 20 ~ 35 years also has a higher incidence of MG in women [8]. The disease status can be unstable during this period, as many patients who initially presented only with ocular symptoms can progress to generalized MG (GMG) within several years after diagnosis [27]. Furthermore, previous research also showed that MG patients with less than two years of disease stability before pregnancy are more likely to experience exacerbation during pregnancy and childbirth [13, 28], which is consistent with our findings.

Although concerns about fetal harm often led to the discontinuation of MG medications during pregnancy, inadequate treatment during pregnancy was also identified as a significant risk factor for pregnancy-related MG relapse. Along with risk factors of younger childbearing age and shorter disease stability duration, these findings indicate the crucial role of stable disease status before and during pregnancy for MG women. Methotrexate, mycophenolate mofetil, and cyclophosphamide are all teratogenic and may increase the risk of miscarriage. Mycophenolate mofetil should be discontinued at least 6 weeks before conception [29]. Cyclophosphamide can lead to amenorrhea and infertility, and thus should be stopped 3 months prior to conception. Methotrexate can be detected in the blood 10 weeks after discontinuation and in the liver 4 months after stopping the drug. Rituximab can cross the placenta and affect the infant’s immune system, necessitating cessation at least 6 months before pregnancy [30]. Pyridostigmine is suitable for use during pregnancy and postpartum as it has minimal impact on fertility. Prednisone is generally considered safe for pregnant women and fetuses [31]. However, case reports indicated an increased risk of cleft lip and palate in the fetus [32], and high-dose corticosteroids during myasthenia crisis (MC) have been associated with obstetric complications [33]. Thus, it should be administered at a safe dose. Calcineurin inhibitors, including cyclosporine and tacrolimus, are generally avoided during pregnancy. Cyclosporine use may lead to preterm birth, and there is insufficient evidence regarding the safety of tacrolimus use during pregnancy. Intravenous immunoglobulin (IVIG) and plasmapheresis can be safely used during pregnancy and delivery.

The thymus gland is pivotal in the pathogenesis of MG. Approximately 75 – 90% of MG patients exhibit abnormal thymus manifestations, including 15% with thymoma and 60% to 80% with thymic hyperplasia [34]. It is recommended for all refractory MG patients with thymoma or thymic hyperplasia to receive thymectomy [35, 36]. For patients planning for pregnancy, the time, safety and benefits of thymectomy are critical clinical decisions. However, the evidence for managing thymic hyperplasia and thymoma is insufficient. Previous meta-analysis indicated that thymectomy before pregnancy significantly improved clinical outcomes for pregnant patients with MG, reducing the worsening rate of MG during pregnancy and postpartum [11]. In our study, 37.17% of patients had thymic hyperplasia and 7.96% had non-invasive thymoma. The regression analysis showed that while the concurrence of thymic hyperplasia increased the risk of MG exacerbation related to pregnancy, thymectomy before pregnancy was identified as a protective factor to reduce the risk. This provides further evidence to support thymectomy efficacy before pregnancy to reduce the exacerbation rate of MG.

Based on these findings, a nomogram prediction model was developed, offering a potential theoretical reference for women with MG to plan pregnancies and reduce the risk of MG relapse related to pregnancy. However, this study has limitations, including the single-centre retrospective design and model selection bias. The risk factors for pregnancy-associated relapse may be different between OMG and GMG, but the sample size is relatively small and, therefore, cannot be assessed at this point. Further prospective studies with larger sample sizes and multi-center data are needed to validate these conclusions.

## Conclusion

The first trimester of pregnancy and the three months following delivery or abortion are high-risk periods for MG relapse. Patients with younger ages, less disease-stable duration, and inadequate treatments during pregnancy are at a higher risk of relapse. Thymic hyperplasia increases the risk of relapse, whereas undergoing thymectomy before pregnancy can reduce the risk.

## Data Availability

The predictive tool implementation of this model is available at https://mgresearch.shinyapps.io/MGpregnancy/. The data that support the findings of this study are available on request from the corresponding author upon reasonable request. For privacy reasons, the data is available from the corresponding author upon request.

## Abbreviations

MG: Myasthenia gravis
AChR: Acetylcholine receptor
MuSK: Muscle-specific tyrosine kinase
LRP4: Low-density lipoprotein receptor-related protein 4
GMG: Generalized myasthenia gravis
OMG: Ocular myasthenia gravis
MC: Myasthenia crisis
IVIG: Immunoglobulin
AUC: Area under curve
BP: Before pregnancy
DP1: 0–3 Months during pregnancy
DP2: 4–6 Months during pregnancy
DP3: 7–9 Months during pregnancy
PP1: 0–3 Months of the postpartum period
PP2: 4–6 Months of the postpartum period
PP3 + 4: 7–12 Months of the postpartum period
